# Nipah Virus–Another Threat From the World of Zoonotic Viruses

**DOI:** 10.3389/fmicb.2021.811157

**Published:** 2022-01-25

**Authors:** Krzysztof Skowron, Justyna Bauza-Kaszewska, Katarzyna Grudlewska-Buda, Natalia Wiktorczyk-Kapischke, Maciej Zacharski, Zuzanna Bernaciak, Eugenia Gospodarek-Komkowska

**Affiliations:** ^1^Department of Microbiology, Ludwik Rydygier Collegium Medicum in Bydgoszcz, Nicolaus Copernicus University in Toruń, Bydgoszcz, Poland; ^2^Department of Microbiology and Food Technology, Jan and Jędrzej Śniadecki University of Technology in Bydgoszcz, Bydgoszcz, Poland; ^3^Department of Biochemistry and Molecular Biology, Wrocław University of Environmental and Life Sciences, Wrocław, Poland

**Keywords:** Nipah, transmission routes, epidemics, diagnosis, health treat, vaccine, pandemic potential

## Abstract

Among the diseases that pose a serious threat to public health, those caused by viruses are of great importance. The Nipah virus (NiV) belonging to the *Paramyxoviridae* family was reported in Malaysia in 1998/1999. Due to its high mortality in humans, its zoonotic nature, the possibility of human-to-human transmission, and the lack of an available vaccine, the World Health Organization (WHO) has recognized it as a global health problem. Depending on strain specificity, neurological symptoms and severe respiratory disorders are observed in NiV infection. In most confirmed cases of NiV epidemics, the appearance of the virus in humans was associated with the presence of various animal species, but generally, bats of *Pteropus* species are considered the most important natural animal NiV reservoir and vector. Consumption of contaminated food, contact with animals, and “human-to-human” direct contact were identified as NiV transmission routes. Due to the lack of vaccines and drugs with proven effectiveness against NiV, treatment of patients is limited to supportive and prophylactic.

## Introduction

One of the consequences of the ongoing Severe Acute Respiratory Syndrome Coronavirus 2 (SARS-CoV-2) pandemic is the increased interest in various aspects related to its origin, virulence mechanisms, potential sources of infection, and modes of transmission. Increasing research to develop an effective epidemic reduction strategy leads to a deeper understanding of the factors that shape its dynamics and the means of controlling it. Paradoxically, the outbreak of a global epidemic may therefore bring beneficial effects in the context of the prevention and monitoring of other infectious agents with epidemiological potential.

Viruses are the cause of diseases that pose a serious threat to public health. Marburg, coronaviruses: Middle East Respiratory Syndrome (MERS) and Severe Acute Respiratory Syndrome Coronavirus 1 (SARS-CoV-1), Human Immunodeficiency Virus (HIV), Hendra, Nipah virus (NiV) and those responsible for Crimean-Congo hemorrhagic fever, Lassa fever, Ebola, Influenza A virus subtype H1N1, Asian highly pathogenic avian influenza (HPAI) A(H5N1) virus or Rift Valley Fever (RVF) caused numerous epidemics in recent years. These epidemics were characterized by high morbidity and mortality, mainly in developing countries in Asia, Africa, and South America (Jones et al., 2008; Aiyar and Pingali, 2020).

The NiV virus, due to its high mortality in humans, its zoonotic nature, the possibility of human-to-human transmission, and the lack of an available vaccine, has been recognized by the World Health Organization (WHO) as a global health problem and included in the list of epidemic threats treated as a priority in research and development activities (R&D Blueprint) (Anderson et al., 2019; World Health Organization [WHO], 2021c). In turn, the Centers for Disease Control and Prevention (CDC) and the National Institute of Allergy and Infectious Diseases (NIAID) classified NiV as category C in the classification of pathogens that pose a terrorist threat (Ochani et al., 2019). The latest information confirms the emergence of the NiV virus in India in September 2021 (World Health Organization [WHO], 2021b).

The study aimed to characterize the NiV virus in the context of its epidemiological potential and to evaluate the effectiveness of previous preventive and intervention measures. Conclusions and observations resulting from such an analysis may constitute suggestions for the development of a scheme of action in the event of an outbreak of an epidemic on a larger than just a local scale.

## Nipah Virus–General Characteristics

The name of the NiV derives from the name of the village of Sungai Nipah (Nipah River Village) in Negeri Sembilan state, Malaysia, where the presence of NiV [IgM antibodies in cerebrospinal fluid (CSF) against Hendra viral antigens] was first confirmed in patient serum samples with symptoms of encephalitis in 1999 (Chua et al., 1999; Parashar et al., 2000). NiV first appeared in Malaysia and Singapore in 1998/1999 (Centers for Disease Control and Prevention [CDC], 1999). The detailed molecular, antigenic and serological analysis made it eligible for the *Paramyxoviridae* family and the genus *Henipavirus*. Paramyxoviruses include many known types of pathogenic viruses, such as the measles virus (MV, genus *Morbillivirus*) or mumps virus (MuV, genera *Rubulavirus*). In turn, the Henipavirus genus, in addition to NiV, also includes three species that are harmless to humans: Cedar virus, Ghanaian bat virus and Mojiang virus, and the highly pathogenic Hendra virus (HeV) (Shoemaker and Choi, 2020). The pathogenic potential of Ghanaian bat virus (Drexler et al., 2012) and Mojiang virus (Wu et al., 2014) are unknown as these have not been isolated, but identified through sequencing data. Sequencing showed a high 80% similarity of NiV and HeV viruses in terms of nucleotide homology (Sharma et al., 2019; Thakur and Bailey, 2019).

Morphologically, NiV resembles other paramyxoviruses: it is a pleomorphic, spherical, or thread-like enveloped virus with a size of 40–1900 nm, containing a single layer of surface protrusions with an average length of approximately 17 nm (Ang et al., 2018; Sharma et al., 2019).

**FIGURE 1:**
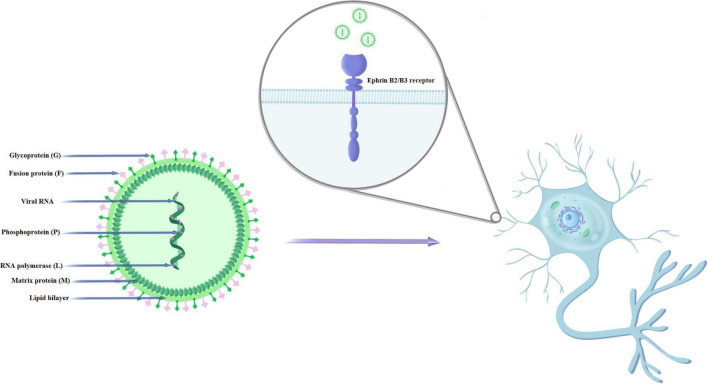
Structure and mechanism of NiV infection of human nerve cells.

Nipah virus has single-stranded RNA of negative polarity. RNA viruses, due to their extremely short generation time and faster evolution, show an increased probability of infection of new host species, although they are already considered the main etiological factors responsible for 25–44% of recently emerging infectious diseases (Harcourt et al., 2001; Carrasco-Hernandez et al., 2017; Devnath and Al Masud, 2021). NiV has six genes, with a genome size ranging from 18,246 to 18,252, depending on the strain (Harcourt et al., 2001; Chakraborty et al., 2019). The NiV genome contains six transcription units that encode the main structural proteins of the virus: nucleocapsid (N), phosphoprotein (P), matrix protein (M), fusion protein (F), attachment glycoprotein (G), and the large protein or RNA polymerase protein (L) (Figure 1). The P gene, in addition to the phosphoprotein, also encodes the NiV proteins C, V, and W, which are responsible for pathogenicity (Wang et al., 2001; Martinez-Gil et al., 2017; Sun et al., 2018; Hauser et al., 2021). Physically related G and F proteins play a key role in binding and fusion to the host cell during the first stage of the viral life cycle. After attaching to the EphrinB2/B3 receptor and entering the cell, the NiV genome is released and replicated. After the transcription catalyzed by the L and P proteins, the viral messenger RNA is translated into the main structural proteins (Hauser et al., 2021). The N protein is responsible, among others, for the proper course of viral replication and transcription. The M protein plays an important role in the final stage of assembling the virion from the genome and proteins, encapsulating and releasing the virus from the host cell (Sun et al., 2018).

Nipah virus mainly enters cells through the fusion of the virus with the cell membrane on the plasma membrane. Cellular expression of NiV glycoproteins can induce cell-membrane fusion (syncytium formation) through the interaction of G and F glycoproteins. The fusion of the virus and host cell membranes (which is pH independent) begins with the binding of G to its host receptor (ephrinB2 or ephrinB3) (Negrete et al., 2005). This is followed by protein conformational changes. F moves from its pre-fusion (PF) conformation to its intermediate hairpin conformation (PHI), causing the fusion peptide to be inserted into the host cell membrane (Aguilar et al., 2010). F then enters the thermodynamically stable six-helical beam (6HB) conformation. The intermediate steps include: the fusion of external flakes (hemifusion), the formation of fusion pores and the widening of the fusion pores (Aguilar et al., 2016). These critical steps in the membrane fusion cascade are necessary for the initiation of infection. Wong et al. (2021) showed that G and ephrinB2 form clusters and that ephrinB2 oligomerization is necessary for F activation. Researchers found no evidence of stable F-G protein complexes before or after activation.

## Nipah Virus Epidemics

### Malaysia

The first human cases of NiV were reported among pig farmers in September 1998 during an epidemic in Malaysia. Initially, they were mistaken for cases of Japanese encephalitis (JE), but both transmission through direct contact with pigs and its occurrence in adults were not typical of infections caused by this infectious agent (Centers for Disease Control and Prevention [CDC], 1999). In May 1999, after the new virus was isolated from the CSF of a patient with Sungai Nipah, NiV was officially recognized as the cause of reported infections (Chua, 2003). Most of the cases were in men who worked with pigs and very few of the patients were young children (Lam and Chua, 2002). Typical symptoms reported by the patients included fever, headache, and decreased consciousness. Depending on the source, the number of cases during the epidemic in Malaysia ranged from 238 to 265 and the number of deaths from 105 to 109, which suggested very high mortality, confirmed during subsequent epidemics (Table 1; Looi and Chua, 2007; Aditi and Shariff, 2019; Ambat et al., 2019).

**TABLE 1:** Morbidity, transmission route, and fatality rate of NiV infections in different NiV epidemic (Ang et al., 2018; Ambat et al., 2019; Sharma et al., 2019; Hauser et al., 2021).

Country	Year/regions	Primary route of transmission	Cases	Death	Fatality rate [%]
Malaysia	1998–1999	Contact with pigs	265	105	39.6
Singapore	1999	Contact with pigs	11	1	9
India	2001 Siliguri	Human-to-human–close direct contact Contact with bats from the *Pteropus* spp.	66	45	68.2
	2007 Nadia		5	5	100
	2018 Kerala		18	17	94.4
	2021 Kerala		1	1	100.0
Bangladesh	2001 Meherpur	Consumption of contaminated fruits and palm sap Person-to-person–close direct contact	13	9	69.2
	2003–2007 Naogaon, Rajbari, Tangail, Kushtia, Natore, Pabna, Thakurgaon		99	78	78.8
	2008–2015 Manikganj, Rajbari, Gaibandha, Nilphamari, Rangpur, Faridpur, Gopalganj, Kurigram, Comilla, Dinajpur, Lalmonirhat, Joypurhat, Naogaon, Natore, Pabna, Magura, Ponchoghor		139	106	76.3
Philippines	2014	Contact with horses Consumption of horse meat	17	9	52.9

### Singapore

Detected in Singapore in March 1999, NiV infections involving the respiratory system and the brain affected slaughterhouse workers who received pigs from Malaysia. Of the 11 cases, 1 was fatal. The median age was 44 years (range 24–66). All patients were men; ten were Chinese, and one Indian (Paton et al., 1999). The epidemic ended with the introduction of a ban on the import of pigs from Malaysia (Chua, 2003; Aditi and Shariff, 2019).

### Bangladesh

In Bangladesh, NiV outbreaks occurred almost annually from 2001 to 2013, mainly in the winter months. They were detected in many parts of the country, some of which appeared several times. Mortality during the first epidemic in 2001 was 69%, and in 2013 it increased to 83%. In total, from April 2001 to March 31, 2012, the number of NiV cases was 209, of which 161 (77%) were fatal (Ambat et al., 2019; Anderson et al., 2019). Gurley et al. (2007) showed that NiV was an ethological factor in the outbreak in the Faridpur district of Bangladesh in April–May 2004. Bangladesh epidemic NiV genomic sequences are presented by Lo et al. (2012). Researchers showed that NiV genomic sequences from patients (two diagnosed in 2008 and three in 2010) constituted a separate genetic group (Lo et al., 2012). Additionally, Lo et al. (2012) proposed the determination of the previously identified NiV strains. Their division assumes that NiV genomic sequences from Malaysia and Cambodia are defined as the M genotype, while the B genotype are sequences obtained from Bangladesh and India. Scientists and doctors were particularly interested in the Bangladesh-specific way of transmitting the virus through the consumption of date palm juice and the transmission between people as a result of direct contact with their secretions of various origins, including the respiratory tract (Aditi and Shariff, 2019).

### India

As in Bangladesh, India’s NiV outbreaks have occurred several times, though not as frequently. The first outbreak of NiV cases was recorded in 2001 in West Bengal, a region across the border from the NiV belt in Bangladesh (Aditi and Shariff, 2019). Confirmed infections and deaths were the highest at the time, at 66 and 45, respectively. The second NiV outbreak occurred in the same region in 2007, with five cases and five deaths (Arankalle et al., 2011). In May 2018, 18 NiV infections were reported in the state of Kerala, mainly characterized by acute respiratory syndrome and encephalitis. At least 17 infected people died then, which means that this NiV outbreak is characterized by the highest mortality, exceeding 90% (Ambat et al., 2019; Plowright et al., 2019). Phylogenetic analysis by Arunkumar et al. (2019) showed that the percent identity of gene fragments of the NiV outbreak that occurred in Kerala, India (May–June 2018) with genotype B NiV (AY988601–Bangladesh 2004) was 97.37–98.64%. The Kerala NiVs were shown to clustered with genotype B viruses but created variations within the clade (Arunkumar et al., 2019). In turn, the phylogenetic analysis by Yadav et al. (2019) based on four human and three fruit bats (*Pteropus medius*) samples from the 2018 outbreak in Kerala, India, found that human NiV was 96.15% similar to the Bangladeshi strain, but 99.7–100% similar to bat virus, meaning bats were the source of the epidemic. Recent reports of NiV cases date from 2019, when a single NiV case was confirmed in the Ernakulam district, culminating in a full recovery of the patient (Sahay et al., 2020).

In 2021, a 12-year-old boy died from the NiV infection on the 5th of September in Kerala’s Kozhikode district. Samples taken after contact-tracing from friends, family members, and health workers were negative (World Health Organization [WHO], 2021b).

### Philippines

The epidemic in the southern part of the Philippines occurred in 2014 and included 17 cases. It was characterized by high mortality, exceeding 80%. The infections were mainly associated with exposure to or consumption of horse meat, and the responsible strain was closely related to the Malaysian strain (Ching et al., 2015; Aditi and Shariff, 2019; Ambat et al., 2019). Fruit bats were the most likely source of horse infection (Ching et al., 2015).

## Symptoms of Nipah Virus Infection

The incubation period for the NiV virus ranges from 4 to 45 days (World Health Organization [WHO], 2021a), with typical symptoms not appearing until after a clinically quiescent period of 4 days to 2 weeks (Ochani et al., 2019; Thakur and Bailey, 2019). Disease caused by NiV infection can be overt or asymptomatic in nature. Cases of such subclinical infections were reported during the NiV epidemics in Malaysia and Singapore, although the percentages for all confirmed cases varied. In Malaysia, it ranged from 8 to 17%, while in Singapore it was even more than 45% (Tan et al., 1999; Parashar et al., 2000; Chan et al., 2002; Chua, 2003; Sharma et al., 2019). In turn, during the epidemic in Bangladesh, asymptomatic infections did not occur or were sporadic (Hsu et al., 2004). They were also rare in India. It confirmed a lower risk of subclinical infections in people who had physical contact with those infected with NiV, but without exposure to their body fluids (Kumar et al., 2019). Recurrence of latent infection has been observed up to months or years after acute infection (Thakur and Bailey, 2019).

Nipah virus infection can affect many major organs, including the brain, lungs, heart, kidneys, and spleen (Thakur and Bailey, 2019). Initial symptoms of NiV resemble flu-like infections, with fever, headache, dizziness, and vomiting (Thakur and Bailey, 2019). However, these symptoms can very quickly turn into an encephalitic syndrome that, in addition to headache and fever, is characterized by serious neurological symptoms. A decrease in consciousness was observed in patients, accompanied by convulsions and visible cerebellar symptoms along with tachycardia (Sharma et al., 2019). In studies conducted in Malaysia, more than 50% of the patients had a low level of consciousness and pronounced brain stem dysfunction (Chua, 2003). However, during the Bangladesh epidemic, the number of infected patients with similar symptoms was much higher, exceeding 90% (Sharma et al., 2019). The result of deep mental disorientation may be a coma appearing after 1–2 days (Singh et al., 2019).

In addition to neurological symptoms, severe respiratory disorders are also observed in NiV infection. Coughs, colds, and shortness of breath, and in extreme cases, acute respiratory distress syndrome, were especially frequently diagnosed during epidemics in Bangladesh and India. These problems are estimated to account for half to two-thirds of the cases. The lower number of patients who declared these symptoms during the epidemics in Malaysia and Singapore was due to differences between the NiV strains responsible for the disease in these regions and the higher frequency of human-to-human transmission confirmed in Bangladesh and India (Goh et al., 2000; Chua, 2003; Sharma et al., 2019; Thomas et al., 2019). Information about the symptoms of NiV infection is summarized in Table 2.

**TABLE 2:** Clinical manifestations of NiV infection.

NiV MY	NiV BD
**Primary influenza-like symptoms:**	
*fever 95%*	*fever 100%*
*headache 75%*	*headache 73%*
*vomiting 32%*	*vomiting 58%*
**Characteristic severe symptoms:**	
• Reduced level of consciousness 72%	• Altered mental status or unconsciousness 90%
• Hyporeflexia 60%	• Areflexia/hyporeflexia 65%
• Encephalitis–segmental myoclonus 32–54%	• Segmental myoclonus–not reported
• Brain stem dysfunction: ∘ abnormal pupils 52% ∘ hypertension 43% ∘ tachycardia 42%	• Respiratory symptoms: ∘ *atypical pneumonia* ∘ *cough 62%* ∘ *respiratory difficulty 69%* ∘ *acute respiratory distress syndrome* 63%
	• Severe weakness 72%

The effects of the virus on the host’s organism depend on the type of strain responsible for the outbreak of a specific infection outbreak. Out of the NiV strains responsible for causing disease in humans tested so far, there are two main strains characterized–the Malaysian strain (NiV-MY) and the NiV Bangladesh (NiV-BD) (Ang et al., 2018; Devnath and Al Masud, 2021). The analyses showed that the NiV genome in Bangladesh and India is 18,252 nucleotides long and is six nucleotides longer than the Malaysian one (Ang et al., 2018; Aditi and Shariff, 2019). In turn, the nucleotide sequences of NiV strains from the Indian regions of Siliguri and Kerala showed 99 and 97% similarity to the strain from Bangladesh, respectively (Pallivalappil et al., 2020). Genetic differences between strains translate into epidemiological effects caused by them. NiV infections in Malaysia have been less severe, with lower mortality and more subclinical cases (Kumar et al., 2019). In Bangladesh and India, genetically similar strains caused a significantly higher number of deaths, although this was also partly related to the low quality of healthcare in these countries and the incidence of new virus outbreaks (Ochani et al., 2019; Sharma et al., 2019; Thakur and Bailey, 2019). The higher infection rate confirmed for the NiV strain from Bangladesh, related to the mode of human-to-human transmission, suggests the possibility of rapid mutation formation (Devnath and Al Masud, 2021). During the epidemic in India in 2001, the clinical symptoms observed in the Siliguri were similar to those of NiV cases diagnosed in neighboring Bangladesh (Chadha et al., 2006). However, the 2018 NiV outbreak in Kerala was characterized by significantly higher mortality than in Siliguri and the occurrence of heart muscle dysfunction in patients–a symptom that had not been reported in previous epidemics (Pallivalappil et al., 2020). The mean disease duration from symptom onset to death was 16 days in Malaysia and only 4–6 days in Bangladesh and India (Ang et al., 2018; Pallivalappil et al., 2020).

## Nipah Virus Transmission

### Animals as a Source of Nipah Virus

Nipah Virus belongs to the group of zoonotic viruses whose source and vector enabling transmission and multiplication are wild and domesticated animals. In some cases of NiV epidemics, depending on the location of the outbreak, the appearance of the virus in humans was associated with the presence of various animal species. Initially, the search for a natural animal NiV reservoir focused on bats in which the HeV, closely related to NiV, was previously detected (Chua, 2003). In Malaysia, the presence of NiV has been confirmed in species *Pteropus hypomelanus*, *Pteropus lylei*, and *Pteropus vampyrus* (Sharma et al., 2019). In India, early research to identify the NiV vector was carried out in insectivorous bats (*Megaderma spasma*), however, its presence was finally confirmed in fruit bats also of the genus *Pteropus*, e.g., *Pteropus giganteus* (Plowright et al., 2019; Thakur and Bailey, 2019). Following the detection of the NiV case in India in 2019, the presence of virus and IgG antibodies was confirmed in bats of the species *P. medius* (Sudeep et al., 2021). The highest degree of sequence similarity of NiV genes from infected bats and Indian patients compared to Malaysia, Cambodia, and Bangladesh clearly suggests that bats were the most likely source of human infection during this epidemic. It could have occurred by eating fruit contaminated with bat saliva or by inhaling an aerosol containing droplets of contaminated urine or saliva (Yadav et al., 2019). Raw date palm juice may also be an important source of the virus, which confirms that the dates of the NiV epidemics in Bangladesh coincide with the palm fruit harvesting and juice production periods (December–May) (Aditi and Shariff, 2019).

The presence of NiV was detected in 9–25% of serologically tested bat samples (antibodies against NiV detected by enzyme−linked immunosorbent assay) from Malaysia, Cambodia, Thailand, and Bangladesh (Sharma et al., 2019). The lack of characteristic clinical symptoms both in wild NiV-positive bats and in individuals intentionally vaccinated with the virus suggests that NiV may have been present in the organisms of these animals for a long time and cause sporadic infections in humans and animals. Only the development of appropriate diagnostic methods, including real-time RT-PCR (TaqMan) tests based on the identification of the specific sequence of the N gene, allowed its identification and monitoring (Thakur and Bailey, 2019). Currently, when looking for evidence of the presence of NiV in bats, but also in other animals, their secretions like saliva, urine, or feces are examined (Looi and Chua, 2007; Sharma et al., 2019; Sudeep et al., 2021).

**FIGURE 2:**
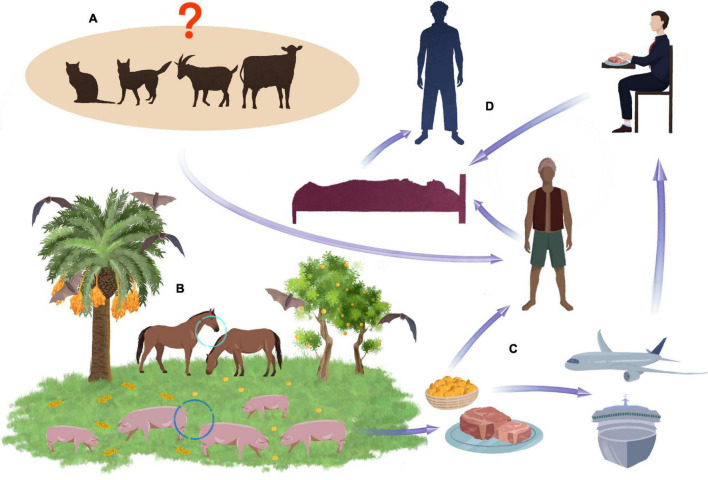
Potential transmission routes of NiV. Pets and some farm animals, such as cows and sheep, are unconfirmed routes of transmission NiV **(A)**. Bats are the natural reservoir of the NiV. When they eat date fruit, the NiV can infect other animals such as pigs and horses, which eat the remains of falling fruit **(B)**. Virus can transmitted to humans through the consumption of the date palm sap or by meat exported all over the world **(C)**. Close contact wit NiV affected human in different situations e.g., in hospital can lead to spread of the virus to other person **(D)**.

Although bats are the primary natural reservoir for the NiV virus, pigs were the source of human infection during the Malaysian epidemic (Looi and Chua, 2007). They act as an intermediate host and become infected by eating fruit contaminated by bats (Figure 2; Thakur and Bailey, 2019). In pigs, NiV causes the syndrome of airway inflammation and encephalitis. The acute form of the disease with fever and cough occurs mainly in animals under 6 months of age. Neurological symptoms include muscle twitching, weakness of the hind legs, and paresis of varying severity (Singh et al., 2019). Despite the incidence of up to 100%, pig mortality from NiV infection is relatively low (Mohd Nor et al., 2000).

In Malaysia, NiV transmission to humans has occurred through close contact with infected pigs (Figure 2). Pig virus isolates in the southern regions of the country had identical gene sequences to those of humans (Abu Bakar et al., 2004). A clear link has been established between infection and the performance of activities related to pig breeding, e.g., drug application, insemination, or disposal of dead animals (Parashar et al., 2000; Thakur and Bailey, 2019). As in Malaysia, cases of NiV infection in Singapore resulted from direct contact with pigs or their feces and involved slaughterhouse workers (Paton et al., 1999; Chan et al., 2002; Aditi and Shariff, 2019).

In Bangladesh and India, the involvement of pigs as the virus vectors and the impact of these animals on the scale of infection have not been observed (Centers for Disease Control and Prevention [CDC], 1999; Thakur and Bailey, 2019). On the other hand, the presence of NiV in people who did not declare contact with pigs was an incentive to search for other potential virus hosts that could pose a threat to humans. Among the various animal species (birds, bats, pigs, and dogs) tested after the epidemic in Bangladesh, no virus was detected in them, and antibodies were found only in bats, which confirms that they could be a source of accidental infection (Hsu et al., 2004). Although NiV has been shown to infect dogs and cats by contact with infected pigs and their secretions, there is no definitive confirmation of their relevance to human infection. The end of the epidemic in Malaysia as a result of the massive destruction of pig herds proves the negligible role of other animals in the spread of NiV (Parashar et al., 2000; Chua, 2003; Mills et al., 2009).

Nipah Virus infection has also been found among hospital workers. NiV nosocomial transmission was reported during the outbreak in Siliguri, India (Chadha et al., 2006). Detection of NiV RNA on a hospital surface indicates that infected patients are transmitting the virus into an environment that could provide an opportunity for NiV transmission to hospital staff. Gurley et al. (2007) showed that environmental samples collected from hospital areas were contaminated with NiV (RT-PCR). The lifetime of NiV on the surface is not known exactly.

### Human-to-Human Transmission

Studies on the spread of NiV during the Bangladesh epidemic led to the conclusion that it was primarily the result of human-to-human transmission of the virus through direct contact with patients and their natural secretions. The primary infection came from a source that is difficult to establish. Probably these were the secretions of the *Pteropus* genus bats (Montgomery et al., 2008). A 2003 study found the presence of the virus within shared households, which could indicate human-to-human transmission (by the respiratory route), but at the same time did not rule out the possibility of external infection. The reason for these doubts was the lack of infections in hospital staff who came into contact with patients (Hsu et al., 2004). However, another study conducted in Bangladesh in 2001–2014 suggests that, in 82 (33.06%) of 248 patients, NiV infection could have been the result of person-to-person transmission of the disease (Nikolay et al., 2020). In contrast, Gurley et al. (2007) found that 92% of patients had close contact with another affected person before becoming ill, and five cases were likely related to secondary and tertiary NiV transmission from person to person. This mode of transmission of the virus was also confirmed during an epidemic in India, both in 2001 in West Bengal and in 2018 in Kerala. Infections occurred in a hospital setting and repeatedly affected healthcare professionals, as well as people visiting patients in hospitals where cases of NiV had been confirmed (Chadha et al., 2006; Thomas et al., 2019).

## Risk Factors

The age and sex of infected people are ranked as basic factors that can determine susceptibility to NiV infection. Studies conducted during the Malaysia epidemic indicate that NiV affected mainly male adults, whose average age ranged from 37 to 44 years (75–81.6%) (Goh et al., 2000; Parashar et al., 2000; Chua, 2003). At the time of the Bangladesh outbreak in early 2004, most NiV-infected patients were young boys under the age of 15. However, it is suspected that the low average age of the infected (11.5 years) was related to the frequency of infection-risk-increasing activities i.e., climbing trees with bats carrying NiV. This factor rather than young age was considered relevant in this case (Montgomery et al., 2008). The analysis of Bangladesh NiV cases between 2001 and 2014 showed that the median age of the patients was 24 years and most of them (64%) were men (Nikolay et al., 2020). However, detailed analyses showed significant differences between the regions of the country. The median age of patients with confirmed or probable NiV infection was 12 years in the Naogaon region, and 38 years in the Meherpur region. A shorter time from the first symptoms of the disease to death (4 days vs. 6 days) was also observed in younger Naogaon patients. These results suggest that both children and adults are susceptible to NiV infection (Hsu et al., 2004). Patients infected with NiV in India in Siliguri province were over 15 years old and the female to male ratio was 1.4:1 (Chadha et al., 2006). Also, the subsequent Kerala epidemic affected mainly men, while the mean age of those infected was 41 years (Thomas et al., 2019).

The high percentage of infections in men observed during various epidemics of NiV may be a direct result of their occupations related to close contact with infected animals or their secretions. In Malaysia, where pigs have been confirmed as the intermediate NiV hosts and contact with them has been the predominant mode of virus transmission to humans, the majority (70%) of infections were in pig farmers and piggery workers (Chua, 2003). Subsequent studies confirmed these data, stating that more than 90% of patients lived or worked on a pig farm, performed work involving exposure to pigs, or had contact with these animals or their urine or feces (Parashar et al., 2000). More than 40% of these people reported the deaths of animals with which they had contact (Goh et al., 2000).

When assessing the degree of risk resulting from contact with pigs, one must also take into account the prevailing method of breeding them in a given region. In Malaysia it is practiced to keep them in large pigsties. Whereas in Bangladesh, which is a Muslim country, the scale of their production is limited, and small herds are usually kept by one person. This limits the possibility of virus transmission both between animals and from animals to humans. It is assumed that, *inter alia*, for this reason, pigs were not involved in the outbreaks in Bangladesh (Looi and Chua, 2007; Aditi and Shariff, 2019). Also in India, pig breeding is carried out on a smaller scale than in Malaysia (Ang et al., 2018).

In addition to people working with animals, healthcare workers are a professional group particularly vulnerable to NiV infection. They were frequently diagnosed with an infection, especially during these epidemics, when the human–human route was the dominant mode of NiV transmission. Working and staying in health care facilities significantly increases the risk of exposure to viruses that enter the environment as a result of coughing and sneezing and are transferred to particles suspended in the air, which is especially dangerous in poorly ventilated rooms. In Bangladesh, the presence of virus RNA on hospital surfaces was proven, but these studies did not conclusively determine the possibility of infection by this route (Gurley et al., 2017). Contact with patients’ body fluids is also a factor that increases the risk of virus transmission to hospital staff (Sayed et al., 2019). In Malaysia, after examining more than 600 health care workers, both exposed and not exposed to contact with patients, no cases were observed, although some of the respondents confirmed needle-stick injuries and exposure of mucous membranes and skin to body fluids (Mounts et al., 1999). During the epidemics that appeared in Bangladesh in 2001–2014, three infections among medical workers were reported (Nikolay et al., 2020). In India, in 2001, the predominant source of infection in the Siliguri region was contact with infected people in healthcare facilities. The 75% of patients were previously staying in a hospital environment including the employees of such facilities (Chadha et al., 2006). During the last confirmed epidemic in the Kerala region in India (2018), hospital transmission of the virus was observed in three hospitals and two health care workers were infected (Thomas et al., 2019). In the context of the increased risk of NiV infection in hospital conditions, which was observed during the epidemic in Bangladesh and India, it is worth emphasizing the importance of regularly confirmed negligence in basic hygiene practices, such as washing hands or the use of personal protective equipment (Chadha et al., 2006; Sayed et al., 2019).

The increased risk of the viral infection also applies to family members caring for patients at home. In the case of countries where NiV outbreaks have occurred so far, this model is supported by the cultural norms (Thakur and Bailey, 2019). Family groups in which family members became infected during caregiving were identified as clusters of infections in India (Thomas et al., 2019). It is also suspected that religious practices specific to some of the countries may influence the spread of the NiV virus. The majority of Bangladeshi Muslims do not eat pork and avoid contact with pigs, which may have influenced the way the virus is transmitted in the country bypassing the intermediate host (Montgomery et al., 2008). On the other hand, the practice of washing corpses, including those who died as a result of NiV infection, before the funeral, consistent with the Islamic funeral ritual, could contribute to the transmission of the virus to family members (Ambat et al., 2019).

The factors determining the scale and intensity of individual epidemics were also climatic phenomena independent of humans, such as catastrophes, which, combined with the progressive anthropogenic degradation of the natural environment, may modify the living conditions of the animal hosts of the NiV virus.

There are hypotheses linking the NiV outbreak with the El Niňo anomaly in 1997–1998 and the drought it caused, which exacerbated the 1997 anthropogenic fires in Indonesia. The resulting dense fog limited the flowering and fruiting of the forest trees. This resulted in the migration of forest fruit bats from their wild habitats to cultivated orchards located near human inhabited places and their farm animals (Looi and Chua, 2007). In Malaysia, the original transfer of NiV from bats to domestic pigs and eventually to humans and other animals was found to occur at a pig farm near which fruit trees were planted. They were to provide a source of additional income and provide shade, and they turned out to be an excellent habitat for NiV-infected bats, whose secretions contaminated the pig feed and water (Chua, 2003; Daszak et al., 2013).

The reduction of the forest area and the progressive urbanization process also destroy the bats’ habitats which, in search of food, move near human settlements. Hunger-induced high stress levels and lowered immunity cause an increase in viremia in their organisms and the virus titer in the urine, semen, and saliva they excrete (Ambat et al., 2019).

## Nipah Virus Diagnostics

Many methods are used in the diagnosis of NiV. Virus isolation in Vero cells, producing cytopathic effects (characterized by syncytia formation and cell death, and an ensuing vasculitis) within 3 days, is used to confirm new NiV foci. Samples from which NiV can be isolated are CSF, respiratory swabs (in viral transport medium), blood, and urine, but all testing procedures must be performed in BSL-4 laboratories (Aditi and Shariff, 2019; Sharma et al., 2019).

Among serological tests based on the detection of IgM and IgG antibodies, ELISA is a simple and inexpensive test (Aditi and Shariff, 2019; Ambat et al., 2019; Sharma et al., 2019). However, it is not 100% specific and may produce false results. Molecular methods provide a higher level of sensitivity and specificity. The polymerase chain reaction (PCR) technique used until recently as a standard has already been replaced by more and more sensitive and specific ones, including conventional reverse transcription PCR (RT-PCR), nested RT-PCR, real-time RT-PCR with the use of intercalating dyes (qPCR), real-time RT-PCR with the use of hydrolysis probes (TaqMan), multiplex bead-based real-time RT-PCR or the RT-LAMP assay. RT-PCR tests for NiV have targeted a highly conserved region of the N, M, or P gene in the viral genome (Guillaume et al., 2004; Ma et al., 2019; Mazzola and Kelly-Cirino, 2019; Ochani et al., 2019; Thakur and Bailey, 2019).

## Treatment

Due to the lack of an effective drug against NiV, the management of patients is limited to supportive and prophylactic treatment (Chakraborty et al., 2019; Sharma et al., 2019). The basic clinical practices in the case of confirmation of NiV infection are maintenance of airway patency, prophylaxis of venous thrombosis, and maintenance of fluid and electrolyte balance (Aditi and Shariff, 2019; Ambat et al., 2019). Mechanical ventilation is used in severe respiratory symptoms. People infected with NiV are also given broad-spectrum antibiotics (Ambat et al., 2019).

Various active substances have been tested in the search for a drug that inhibits NiV proliferation. However, the effectiveness of ribavirin administered during the epidemic in Malaysia is debatable, as is the effectiveness of acyclovir used in Singapore (Ambat et al., 2019; Sharma et al., 2019; Devnath and Al Masud, 2021). The antimalarial drug chloroquine showed effectiveness in inhibiting NiV in cell cultures, which could not be confirmed in animal models (Aditi and Shariff, 2019; Ambat et al., 2019; Ochani et al., 2019). Promising results were observed after the application of the drug Favipiravir (T-705) and the monoclonal antibodies m102.4 in animals (Sayed et al., 2019; Singh et al., 2019; Devnath and Al Masud, 2021). The monoclonal antibody m102.4 is in phase I human trials (Playford et al., 2020). Eighty-six treatments related adverse events were reported, with similar rates in the placebo and treated groups. No deaths have been recorded (Playford et al., 2020). Lo et al. (2020) evaluated the *in vitro* antiviral activity of GRFT (Griffithsin) and its synthetic trimeric tandemer (3mG) against NiV and other viruses from four virus families. Researchers demonstrated that a preliminary *in vivo* evaluation of oxidation-resistant GRFT (Q-GRFT) showed significant protection against a lethal NiV challenge in golden Syrian hamsters. GRFT is a high mannose oligosaccharide-binding lectin that has shown a broad spectrum *in vivo* activity against viruses (Lo et al., 2020).

## Vaccines

Clinical trials with vaccines to protect against NiV are limited due to the lack of new viral outbreaks. The effectiveness of potential preparations is tested in animal models. To date, research has included over 10 vaccines based on viral vectors, mRNA, recombinant protein subunits, or virus-like particles (Broder et al., 2016; Amaya and Broder, 2020). The most studied so far is the subunit vaccine based on soluble recombinant Hendra G-glycoprotein (HeV-sG), also inducing a cross-immune response against NiV. It has been proven to be completely effective in protecting against NiV MY, NiV BD, and HeV in horses, cats, ferrets, and non-human primates, while it did not show effectiveness in pigs (Mungall et al., 2006; McEachern et al., 2008; Bossart et al., 2012; Pallister et al., 2013; Middleton et al., 2014; Mire et al., 2014). The only vaccine officially approved and registered by the Australian Pesticides and Veterinary Medicines Authority (APVMA) vaccine is Equivac produced by Zoetis, Inc. It is used in the prophylactic treatment of horses (Aditi and Shariff, 2019; Ambat et al., 2019; Ochani et al., 2019; Geisbert et al., 2021). Geisbert et al. (2021) conducted two studies on this NiV vaccine. The first one showed that a single dose of vaccination was effective, and the second one showed that the period of 7 days after the first dose was effective in protecting African green monkey (AGM) from the debris form of NiV. Studies conducted in an animal model similar to the human organism–the AGM model confirm the effectiveness of a single dose of this vaccine, which may constitute the basis for its potential use in the event of sudden outbreaks of NiV infection (Geisbert et al., 2021).

Animal studies have also shown the efficacy of recombinant vesicular stomatitis viruses (rVSV recombinant vesicular stomatitis viruses, rMV recombinant measles virus) (Yoneda et al., 2013; Debuysscher et al., 2014; Ang et al., 2018). Yoneda et al. (2013) conducted a study of the effectiveness of recombinant rMV in hamsters and AGM. Researchers demonstrated an increase in anti-NiV antibodies in the serum of hamsters within 3 weeks after inoculation. The hamsters showed no signs of disease and no mortality. Immunization with AGM with two doses of the vaccine protected the monkeys from clinical signs of disease, and levels of antibodies to the NiV G protein were induced. Also, Debuysscher et al. (2014) assessed the recombinant rMV expressing NiV glycoproteins (G or F) or nucleoprotein (N) in a Syrian hamster model. The results were promising as even the first dose of vaccination produced a high humoral immune response (Debuysscher et al., 2014).

The search for new vaccines is constantly ongoing. A vaccine designed by Soltan et al. (2021) is a multi-strain vaccine based on the highest-ranked CTL, HTL, and BCL epitopes from selected NiV proteins. The vaccine design was analyzed for allergenicity and toxicity, physicochemical features, antigenicity, and solubility (computational analysis). The designed vaccine was shown to be non-toxic and non-allergenic (Soltan et al., 2021). However, experimental trials are required to prove the practical effectiveness of this potential vaccine construct. Another attempt to design a vaccine against NiV was made by Loomis et al. (2020). Researchers assessed antigenicity and structural integrity protein subunit vaccines (F and G proteins, stabilized pre-F and G to form a chimeric protein) using kinetic binding assays, electron microscopy and other biophysical properties. Vaccine efficacy was assessed in a mouse model. Mice immunized with all the pre-F designs showed similar levels of pre-F and post-F binding. All mice immunized with multimeric G forms induced only G-specific antibody responses, while mice immunized with pre-F/G or pre-F + G chimeras generated antibody responses directed against both F and monomeric G proteins. Loomis et al. (2020) confirmed that the NiV G glycoprotein is an effective vaccine immunogen and it is feasible to design both pre-F and G as a single vaccine construct which confers greater diversity in responses to antigenic sites (Loomis et al., 2020). Loomis et al. (2020) confirmed that the F NiV protein pre-fusion induces stronger neutralizing activity than post-fusion F, thus confirming the importance of stabilizing the pre-fusion conformation for increasing immunogenicity. In another study by Loomis et al. (2021), an attempt was made to stabilize the fusion protein (F) in its pre-fusion trimeric (pre-F) conformation to increase expression and immunogenicity using design principles based on protein structure. Loomis et al. (2021) have shown that Pre-F and G induce potent neutralizing antibody responses as mRNA vaccines.

Vaccinations are an important part of the fight against the epidemic. Continued work on new vaccines against NiV is necessary, especially in view of clinical trials.

## Prevention

Due to the limited possibilities of effective treatment and the lack of a vaccine, it seems justified to focus the efforts of scientists and institutions responsible for monitoring epidemiological threats on preventing NiV emergence and their effective supervision.

Preventive strategies to limit the appearance of new and the spread of already initiated epidemic outbreaks relate primarily to avoiding direct contact with the hosts of the virus (fruit bats and pigs) and their secretions and avoiding consumption of contaminated food. On the one hand, it is recommended to carefully check and wash the fruit of the trees where bats live. On the other hand, procedures are implemented to limit their access to places and vessels used to collect date palm juice (bamboo skirt method) (Ambat et al., 2019; Sharma et al., 2019). It is also recommended not to plant fruit trees that attract bats near the piggery (Ang et al., 2018). During work requiring direct contact with farm animals, especially during slaughter and disposal procedures, appropriate protective clothing is required (Aditi and Shariff, 2019).

Implementation of actions raising public awareness of the risks associated with the outbreak of the virus and the importance of preventive measures is another factor that may help to the risk of the new outbreak. Local television, radio channels, and printed media informed local societies, for example, on the dangers of drinking contaminated date palm juice, recommending that its consumption should be limited (Aditi and Shariff, 2019). Similar attempts to eliminate the consumption of fresh juices turned out to be ineffective, as they contradicted the prevailing social and cultural norms (Ang et al., 2018).

An important preventive measure that can effectively reduce the scale of the spread of NiV is to prevent the spread of the virus through direct human-human contact. Washing hands, disinfecting with 70% ethanol, wearing gloves and other protective equipment, and avoiding direct contact with body fluids are standard rules for caring for a person infected or just suspected of having NiV infection (Sharma et al., 2019). In a hospital setting, sharing food and bed between patients is unacceptable.

It is important to evaluate the disinfection efficiency against NiV. Both chemical and physical methods. Like other paramyxoviruses, NiV is easily deactivated by soaps, detergents and many disinfectants. Sodium hypochlorite has been recommended for disinfection of pig farms in Malaysia (Spiclera, 2016). Eickmann et al. (2020) demonstrated an effective reduction of NiV infectivity in platelet and plasma concentrates using UV-C and MB/light (increasing doses of visible light). Treatment with 0.2 J cm^2^ UV-C reduced the infectivity of NiV (≥4.3 log) to the limit of detection (LOD) in platelet concentrates, and treatment with MB (120 J/cm^2^) reduced NiV (≥2.7 log) to LOD in the plasma.

Transmission of infections to healthcare professionals can be reduced by ensuring access to hand hygiene facilities, regular and appropriate use of personal protective equipment, and isolating patients with meningitis. It is of particular importance to follow appropriate procedures during funeral ceremonies–wearing gloves and masks, washing hands thoroughly with soap and water immediately after contact with the corpse (Ambat et al., 2019).

In the regions along the NiV belt, surveillance is also carried out to enable early detection of virus outbreaks, analysis of its strains, and monitoring of the relationship between environmental factors and the dynamics of epidemic development. For this purpose, specialized research teams are established to identify suspected cases of infection in humans and analyze the possibility of the emergence of new species of potential reservoir hosts of the virus responsible for its transmission to humans (Aditi and Shariff, 2019; Ambat et al., 2019).

## Nipah Virus–Pandemic Potential

The situation of the global COVID-19 pandemic, which has resulted in the deaths of nearly 5 million people, encourages scientists to conduct research that will stop the spread of the virus, reduce its effects and help to develop an effective strategy for recognizing, preventing, and managing the emergence of other pathogens with epidemic potential.

One of the features indicating the viral pandemic potential is the rapid and effective transmission of the pathogen from person to person, especially in the absence of immunity in exposed people. This route of NiV transmission was confirmed to be extremely important during the epidemics in Bangladesh and India. Infection may occur as a result of direct contact with an infected person or their secretions, and an additional risk is posed by the possibility of virus transmission with saliva particles in patients with severe respiratory symptoms and accompanying cough (Luby et al., 2009). Many studies indicate that respiratory tract secretion is the main source of infection in accompanying patients and confirm the relationship between virus transmission through this route and the degree of manifestation of symptoms of advanced changes in the respiratory system (Luby, 2013).

The airborne route is also the main mode of transmission of the SARS-CoV-2 virus, which results in the introduction of general orders to apply appropriate protective measures in the pandemic society (Dhand and Li, 2020).

The high mortality characteristic of NiV is a factor that can limit the spread of a virus that does not infect another due to the death of the host. However, it is worth taking into account that the current NiV outbreaks were usually detected in sparsely populated villages (Devnath and Al Masud, 2021). It can be assumed that in the event of an epidemic in a highly populated place, the increase in infections rate could be much higher, especially since, according to WHO, the incubation period for NiV in extreme cases may last 45 days. People-to-people contacts are favored by the growth of the human population globalization, trade, or contemporary travel patterns (Carrasco-Hernandez et al., 2017). Taking into account the risk associated with the appearance of a certain percentage of subclinical cases, the rank of NiV as a potential source of another global epidemic increases.

Amidst the known and tested strains of NiV so far, differences have been observed in transmission methods, the symptoms type and severity, and finally the size of genomes confirmed by molecular analyses. Indirectly, this indicates the need to include the risk of the emergence of mutations, e.g., facilitating the human-to-human viral transmission, in the assessment of the NiV pandemic potential. Such suggestions were made when sequencing of a NiV genome fragment from a Filipino patient (2014) showed a high degree of similarity to the Malaysian strain. However, while in the case of the epidemic in Malaysia no human-to-human transmission was recorded, this possibility has already been confirmed in the Philippines (Ching et al., 2015). RNA viruses, due to their commonly observed high rate of nucleotide substitutions, poor ability to correct mutation errors, and the resulting adaptability to new hosts, are regarded as extremely dangerous and require close supervision (Jones et al., 2008).

In addition to human-human contact, NiV infection can occur through contact with animals. The primary reservoir of NiV is bats, which are likely to have also been a source of COVID-19 infections in humans (Zhou et al., 2020). The detection of henipaviruses in bats in China and West Africa may suggest that the diseases they cause will also appear outside of Asia and Australia (Halpin and Rota, 2014; Kasloff et al., 2019). The range of NiV hosts, unlike other paramyxoviruses, is surprisingly wide, resulting from the virus binding to the ephrin B-2 and B-3 receptors, common in mammalian epithelial cells (Luby, 2014). The possibility of domestic and farm animals infection creates additional opportunity of NiV transmission to humans (Ang et al., 2018). Recent studies show that pigs excluded as a source of the virus during an epidemic in Bangladesh are susceptible to NiV-B infection without evidence of clinical disease. The presence of an infectious virus in the nasal wash may indicate the possibility that asymptomatically infected pigs infect susceptible animals and spread the virus (Kasloff et al., 2019).

## Conclusion

Drawing conclusions from the COVID-19 pandemic, we must be prepared for the fact that any zoonotic virus, especially one with the ability to human-to-human transmission, can be very dangerous and contribute to a global pandemic. An important argument justifying the concerns about the emergence of NiV in the human population is the lack of vaccines and drugs with proven effectiveness. Attempts to develop them do not bring the expected results, and in combination with the problems in the healthcare efficiency revealed during the ongoing COVID-19 epidemic, both in the poorest and highly developed countries, they can result in serious consequences on a global scale (Ang et al., 2018; Gurley et al., 2020).

## Author Contributions

KS, JB-K, and KG-B: conceptualization. KG-B and MZ: formal analysis. KS, JB-K, and NW-K: writing—original draft preparation. KS, KG-B, and MZ: writing—review and editing. ZB, KG-B, and NW-K: visualization. KS and EG-K: supervision. KS and JB-K: project administration. All authors have read and agreed to the published version of the manuscript.

## Conflict of Interest

The authors declare that the research was conducted in the absence of any commercial or financial relationships that could be construed as a potential conflict of interest.

## Publisher’s Note

All claims expressed in this article are solely those of the authors and do not necessarily represent those of their affiliated organizations, or those of the publisher, the editors and the reviewers. Any product that may be evaluated in this article, or claim that may be made by its manufacturer, is not guaranteed or endorsed by the publisher.
